# Transcriptomic Analysis Reveals Novel Regulators of the Scots Pine Stilbene Pathway

**DOI:** 10.1093/pcp/pcad089

**Published:** 2023-08-09

**Authors:** Tanja Paasela, Kean-Jin Lim, Mirko Pavicic, Anni Harju, Martti Venäläinen, Lars Paulin, Petri Auvinen, Katri Kärkkäinen, Teemu H Teeri

**Affiliations:** Department of Agricultural Sciences, Viikki Plant Science Centre, University of Helsinki, PO Box 27, Helsinki 00014, Finland; State Key Laboratory of Subtropical Silviculture, College of Forestry and Biotechnology, Zhejiang A&F University, Lin’an District, Hangzhou, Zhejiang 311300, China; Oak Ridge National Laboratory, Biosciences Division, 1 Bethel Valley Rd, Oak Ridge, TN 37830, USA; Production Systems Unit, Natural Resources Institute Finland (Luke), Vipusenkuja 5, Savonlinna 57200, Finland; Production Systems Unit, Natural Resources Institute Finland (Luke), Vipusenkuja 5, Savonlinna 57200, Finland; Institute of Biotechnology, University of Helsinki, PO Box 56, Helsinki 00014, Finland; Institute of Biotechnology, University of Helsinki, PO Box 56, Helsinki 00014, Finland; Production Systems Unit, Natural Resources Institute Finland (Luke), Paavo Havaksentie 3, Oulu 90570, Finland; Department of Agricultural Sciences, Viikki Plant Science Centre, University of Helsinki, PO Box 27, Helsinki 00014, Finland

**Keywords:** 4-Coumarate-CoA ligase, Cycloheximide, Pinosylvin, Regulation, RNA-seq, Secondary metabolism, Stilbene synthase, Transcription factor, Transcriptomics, UV-C

## Abstract

Stilbenes accumulate in Scots pine heartwood where they have important roles in protecting wood from decaying fungi. They are also part of active defense responses, and their production is induced by different (a)biotic stressors. The specific transcriptional regulators as well as the enzyme responsible for activating the stilbene precursor cinnamate in the pathway are still unknown. UV-C radiation was the first discovered artificial stress activator of the pathway. Here, we describe a large-scale transcriptomic analysis of pine needles in response to UV-C and treatment with translational inhibitors, both activating the transcription of stilbene pathway genes. We used the data to identify putative candidates for the missing CoA ligase and for pathway regulators. We further showed that the pathway is transcriptionally activated by phosphatase inhibitor, ethylene and jasmonate treatments, as in grapevine, and that the stilbene synthase promoter retains its inducibility in some of the tested conditions in Arabidopsis, a species that normally does not synthesize stilbenes. Shared features between gymnosperm and angiosperm regulation and partially retained inducibility in Arabidopsis suggest that pathway regulation occurs not only via ancient stress-response pathway(s) but also via species-specific regulators. Understanding which genes control the biosynthesis of stilbenes in Scots pine aids breeding of more resistant trees.

## Introduction

Stilbenes are important secondary metabolites for both the induced and constitutive defense systems in many plant species (Chong et al. 2009). The stilbenes of Scots pine (*Pinus sylvestris* L.) consist mainly of pinosylvin (3,5-dihydroxystilbene) and its monomethylether (3-methoxy-5-hydroxystilbene). Additionally, a minute amount of dimethyl ether (3,5-dimethoxystilbene) can be found in pine heartwood (Venäläinen et al. 2004, Hovelstad et al. 2006). The developmentally regulated heartwood stilbenes are important determinants of heartwood decay resistance, and especially, the concentration of heartwood pinosylvin has high heritability. In addition, there is a wide variation between individuals in their capacity to produce and accumulate stilbenes in their heartwood (Harju and Venäläinen 2006, Leinonen et al. 2008, Belt et al. 2021). The pathway is also stress responsive, and there is a genetic correlation between stress-induced stilbene content in seedlings and stilbene content in the heartwood of their mother trees (Harju et al. 2009). The offspring of individuals having the highest amount of pinosylvin in their heartwood produce more stilbenes as a response to wounding of the xylem (Harju et al. 2009). The regulation of the pine stilbene pathway and the biosynthetic enzymes is largely unknown, unlike in the angiosperm stilbene-producing plant grapevine. Understanding the causative genetic differences in stilbene production between individuals aids the breeding of more durable heartwood and more stress-tolerant trees (Harju and Venäläinen 2006, Valletta et al. 2021).

The biosynthetic pathway leading to stilbene formation consists of four enzymes. Stilbene synthase (STS) (Schwekendiek et al. 1992) and pinosylvin *O*-methyltransferase (PMT) (Paasela et al. 2017) are characterized, but the pathway-specific cinnamate-activating CoA ligase is still unknown. In comparison to angiosperm and spruce stilbenes, pine stilbenes are structurally different deriving from cinnamoyl-CoA instead of 4-coumaroyl-CoA. In Aaron’s beard (*Hypericum calycinum*) and petunia (*Petunia hybrida*), cinnamate is activated by cinnamate-CoA ligase (CNL) in the biosynthetic pathway to benzoic acid and its derivatives (Gaid et al. 2012, Klempien et al. 2012). These enzymes have low sequence similarity with 4-coumarate-CoA ligase (4CL) enzymes. Cinnamate can also be activated by specific 4CL variants like in *Rubus idaeus* and *Arabidopsis thaliana* (Ehlting et al. 1999, Kumar and Ellis 2003, Costa et al. 2005). It is not known either if there are stilbene pathway–specific phenylalanine ammonia lyases (PAL), the fourth enzyme of the pathway, or is this step shared with flavonoid and/or lignin pathways.

We have previously studied the induction of stilbene biosynthesis under the developmental context of heartwood formation (Lim et al. 2016) and in response to mechanical wounding (Lim et al. 2021). It has also been previously shown that the stilbene pathway in seedlings is strongly induced by UV-C treatment (Schoeppner and Kindl 1979, Preisig-Müller et al. 1999). The UV spectrum is divided into UV-A (315–400 nm), UV-B (280–315 nm) and UV-C (100–280 nm), of which UV-C represents the shortest wavelength region (Rai et al. 2021). The most damaging wavelengths, including UV-C, are completely absorbed by the atmosphere from solar UV radiation, but it can be used as an experimental tool to study, for instance, programmed cell death (PCD), DNA damage and repair mechanisms (Gallego et al. 2000, Gentile et al. 2003, Danon et al. 2004, Gao et al. 2008, Rai et al. 2021), as well as stilbene pathway activation and regulation (Vannozzi et al. 2012, Xi et al. 2014, Suzuki et al. 2015). When used experimentally, UV-C produces a strong reactive oxygen species (ROS) burst mainly from chloroplasts and mitochondria (Gao et al. 2008). UV-C responses are mediated by nonspecific stress-signaling pathways originating from ROS, DNA damage and wound/defense signaling involving ethylene, jasmonic acid (JA) and salicylic acid (SA) (Jenkins 2009, Nawkar et al. 2013).

The effect of UV-C in activating the stilbene pathway has been studied before in more detail in grapevine (*Vitis vinifera* L.) (Vannozzi et al. 2012, Xi et al. 2014, Suzuki et al. 2015), and UV-C transcriptomic data have been utilized to identify specific regulators of grapevine stilbene biosynthesis (Höll et al. 2013). Several R2R3-MYB and WRKY transcription factors (TFs) have been identified as STS regulators in different grapevine species in response to stress or developmental cues (Höll et al. 2013, Wong et al. 2016, Vannozzi et al. 2018, Jiang et al. 2019, Wang et al. 2019). Some of these regulators have been shown to act as activators and some as repressors of STS gene expression. For instance, the R2R3-MYB TFs VvMYB14 and VvMYB15 were shown to mediate *VvSTS* gene induction and stilbene production, both after stress induction (wounding, UV-C and pathogen infection) and during fruit development at the onset of ripening (Höll et al. 2013). A WRKY TF, VvWRKY03, is upregulating *STS* gene expression by acting together with VvMYB14 (Vannozzi et al. 2018). VvWRKY8 is repressing the expression of stilbene biosynthesis by binding the VvMYB14 activator and this way preventing its action (Jiang et al. 2019). Different regions of the STS gene promoters are responsible for pathogen and ozone responsiveness, and ozone and ethylene activate *VvSTS* expression via separate signaling pathways (Grimmig et al. 1997, Schubert et al. 1997).

Protein synthesis inhibitors like cycloheximide (CHX) provide widely used and useful tools for identifying primary response genes (PRGs) that do not require de novo protein synthesis but are regulated by preexisting TFs and labile repressors. The kinetics of the initiation of transcription vary among the PRGs, and they can further be categorized as PRGs and delayed PRGs (Tullai et al. 2007). Conversely, secondary response genes (SRGs) are dependent on the synthesis of new TFs after the stimulus and cannot be activated if the translation is inhibited (Tullai et al. 2007, Bahrami and Drabløs 2016).

In this study, the transcriptional response to UV-C of pine needles was analyzed to discover regulators and missing enzymes of the Scots pine stilbene biosynthetic pathway. We discovered several TFs and two 4CL-encoding genes that were co-expressed with *PsSTS*. However, their involvement in the stilbene pathway needs to be studied in detail with additional experiments. Since the expression of *PsSTS* was induced relatively fast after the UV-C exposure, we tested if the expression would be independent of de novo protein synthesis using CHX. CHX did not prevent the accumulation of *PsSTS* transcripts, and the treatment alone was able to activate the transcription. Transcriptional responses to CHX were studied using RNA-seq as well. Such large-scale transcriptional analysis of UV-C or CHX responses has not been performed in gymnosperm species before. Also, the effect of CHX in stilbene pathway activation in grapevine is not known. It is known that stilbene biosynthesis in pine is transcriptionally regulated, but the mechanisms and components of the signaling pathways under different inductive conditions are unknown. In grapevine, it has been shown that the plant hormones JA, its volatile derivative methyl jasmonate (MeJA) and ethylene activate the STS-encoding genes (Grimmig et al. 1997, Tassoni et al. 2005, Belhadj et al. 2008). The tyrosine phosphatase inhibitor, sodium orthovanadate, is another known inducer of *VvSTS* (Tassoni et al. 2005). In this study, we showed that the stilbene pathway is activated in pine in response to phosphatase inhibitors and plant hormones, as it is in grapevine. In addition, pine STS promoter was able to activate the expression of a reporter gene in Arabidopsis, a species that does not naturally produce stilbenes, in response to UV-C exposure and phosphatase inhibitors, but not as a response to plant hormone treatments. The shared features between gymnosperms and angiosperms regulation indicate that STS regulation occurs not only via ancient stress-response pathway(s) but also via species-specific regulators.

## Results

### Induction of the stilbene pathway as a response to UV-C and CHX treatments

**Fig. 1:**
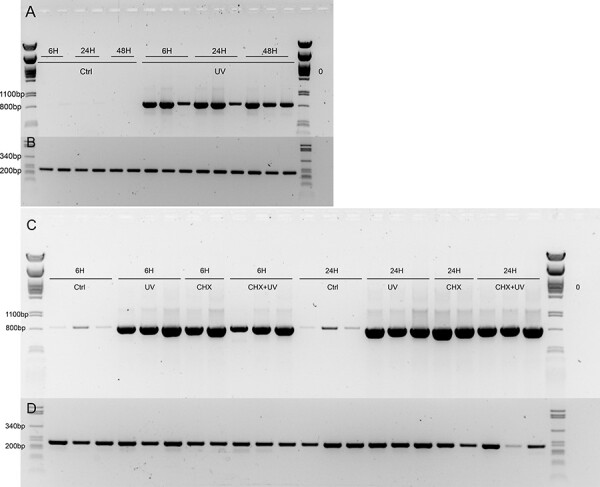
(A) The expression of *PsSTS* was followed with RT-PCR at 6, 24 and 48 h after the onset of UV-C treatment. The expression of *PsSTS* was evident at all the analyzed time points but not at the untreated control samples. The expected size of the *PsSTS* fragment is 853 bp. (B) The expression of housekeeping gene *actin* was used as a control and was analyzed from the same samples. (C) The expression of *PsSTS* was analyzed 6 and 24 h after the onset of UV-C treatment. Plants were treated overnight with 10 μM of CHX prior to the UV exposure. (D) The expression of housekeeping gene *actin* was used as a control.

Six-week-old Scots pine seedlings were treated with UV-C, and the expression of the transcript encoding the key enzyme of the stilbene pathway, *PsSTS*, was followed for 48 h with semi-quantitative RT-PCR (**Fig. 1A**, **B**, **Supplementary Fig. S3**). *PsSTS* was strongly induced already at 6 h after the treatment (**Fig. 1A**, **Supplementary Fig. S3**). Pinosylvin was detected from the needles 24 h after the treatment (**Supplementary Fig. S1**).

Since the expression of *PsSTS* was induced relatively fast after the UV-C exposure, we tested if the expression would be independent of de novo protein synthesis. The translation was inhibited using an overnight treatment of CHX prior to the UV-C treatment. Inhibition of protein synthesis did not prevent the accumulation of *PsSTS* transcripts, and in fact, CHX treatment alone was able to activate *STS* gene expression (**Fig. 1C**, **D**). Based on the incorporation rate of radioactive methionine in needle proteins, CHX treatment inhibited translation by 94%. Another protein synthesis inhibitor, anisomycin, was also able to activate *PsSTS* gene expression (**Supplementary Fig. S2**).

### Transcriptional changes in response to UV-C treatment

Transcriptomes of needles from 6-week-old seedlings treated with UV-C and sampled from four Scots pine families at 2, 6 and 24 h after the onset of the treatment were sequenced using SOLiD platform and compared to untreated control (**Supplementary Fig. S3**). In response to UV-C treatment, 6,131 tentative consensus (TC) sequences in the Pinus EST collection (The Dana-Farber Cancer Institute gene index databases, 2014) showed differential expression [false discovery rate (FDR) <0.05] in at least one of the time points when compared to control samples, based on analysis by edgeR (**Supplementary Table S1**). Mapping was performed against the EST collection instead of a Trinity assembly of our own data to observe the expression of genes that were not present in our data, as described in our previous studies (Lim et al. 2016, 2021). The similarity of the replicate samples was tested using Multidimensional Scale Plot (MDS) in edgeR. The replicate samples in each time point were grouped together. The highest dispersion between the replicates is seen in samples collected after 2 h of UV-C or CHX treatments (**Supplementary Fig. S4**).

**Fig. 2:**
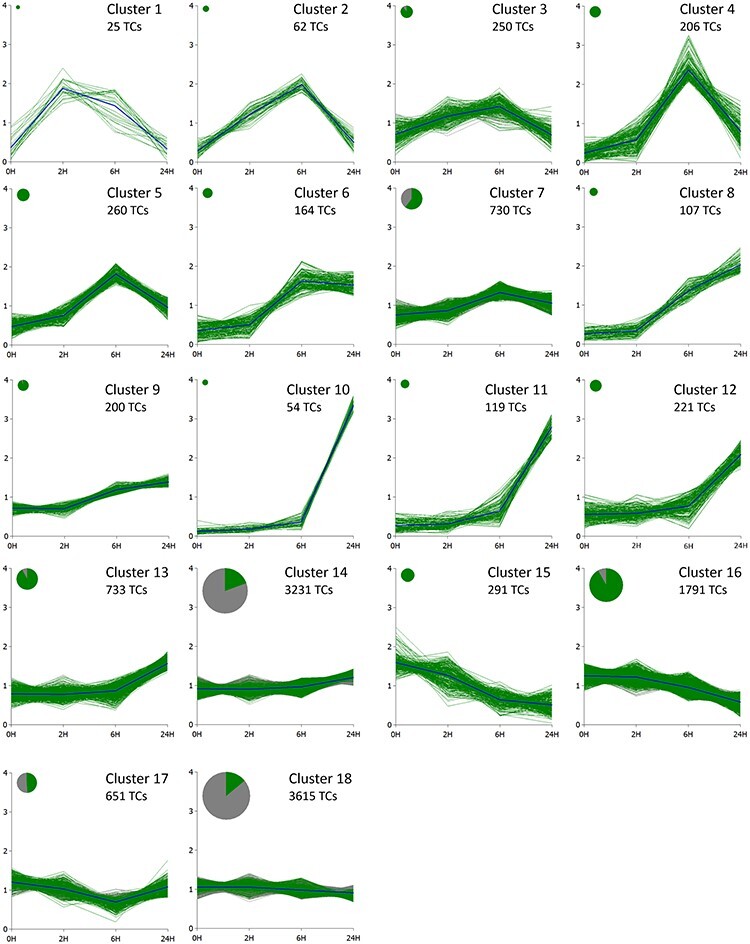
Transcripts from UV-C treatment data were grouped by the Bayesian hierarchical clustering algorithm *SplineCluster* (Heard et al. 2006). All TCs that obtained at least eight CPM in at least four libraries are shown. The transcripts that have statistically significant (FDR < 0.05) differences in expression levels compared to the control are shown in color. The area of the disc is proportional to the number of TCs in the cluster. 0H, control; 2H, 2 h; 6H, 6 h and 24H, 24 h.

To get a better view of which processes were upregulated and which were shutting down in response to UV-C treatment, hierarchical clustering was used to capture specific expression patterns of transcripts. A closer look was taken at different secondary metabolite pathways to see if they behaved in a similar manner as the stilbene pathway. Clustering was also utilized to discover specific PAL- and 4CL-encoding genes or transcriptional regulators that would fall in the same clusters as *STS* and *PMT2* and could be involved in stilbene biosynthesis. Sequence data were mapped against Pinus EST collection, and 12,709 transcripts that achieved at least eight counts per million (CPM) in at least four libraries were subjected to *SplineCluster* analysis, which uses a Bayesian hierarchical clustering algorithm (Heard et al. 2006). Transcripts were grouped into 18 clusters according to their expression profiles (**Fig. 2**, **Supplementary Table S2**), and Gene Onthology (GO) enrichment analysis was performed for each cluster and is shown in **Supplementary Table S3**. Based on the analysis, four different expression patterns could be distinguished. In the first pattern, in Clusters 1–7, the expression peaked either 2 or 6 h after the treatment and then declined and in some cases returned to the control level. In the second pattern, in Clusters 8–14, transcription was activated after the treatment, but the peak of the expression was beyond the time points measured. In the third pattern, Clusters 15 and 16, the expression of transcripts was downregulated. Cluster 18 formed the final pattern where the expression pattern was flat. In upregulated clusters, GO terms such as stress, stimulus and signaling were enriched, and these included transcripts for counteracting the oxidative stress such as glutathione-*S*-transferase, thioredoxin H-type and alternative oxidase. Photosynthesis and chloroplast-related terms were enriched in downregulated clusters. Another enriched group of genes downregulated in response to UV-C stress was those involved in cell wall modifications and the core histone proteins.

### Transcriptional responses of secondary metabolite pathways to UV-C stress

The expression of stilbene pathway–encoding genes was upregulated in response to UV-C treatment. The transcripts were found in different clusters. Full-length PsSTS and PsPMT2 enzymes are encoded by TCs TC154538 and TC166778, respectively (Schanz et al. 1992, Paasela et al. 2017). Both were found in Cluster 8, where genes were upregulated but did not reach the peak expression in 24 h. Most of the PAL- and 4CL-encoding transcripts followed the first pattern where the expression started to decline after the initial induction. They were present in Clusters 3–7. Some could also be found in Clusters 13 and 14 that showed mild upregulation without a decline in the expression.

The mevalonate (MVA) pathway genes, such as 3-hydroxy-3-methylglutaryl coenzyme A reductase (HMGR), were strongly upregulated by UV-C exposure and were present in Clusters 3, 4 and 6. The MVA pathway is important for the biosynthesis of essential terpenoid products such as sterols, sesquiterpenes, triterpenes, cytokinins and brassinosteroids (Rodríguez-Concepción and Boronat 2002). The sesquiterpene biosynthetic enzyme *E*,*E*-*α*-farnesene synthase was strongly upregulated in Cluster 4. In contrast, genes encoding enzymes for the core chloroplastic isoprenoid-producing pathway, the methylerythritol 4-phosphate (MEP) pathway and enzymes downstream of MEP involved in monoterpene and diterpene resin acid biosynthesis were downregulated.

Lignin biosynthesis pathway genes such as cinnamoyl-CoA reductase (CCR), caffeoyl-CoA *O*-methyltransferase (CCoAOMT) and hydroxycinnamoyl-CoA:shikimate hydroxycinnamoyltransferase (HCT) were induced in response to UV-C treatment moderately, 2- to 5-fold, and followed the first expression pattern.

The expression of chalcone synthase, the key enzyme of the flavonoid pathway, or other enzyme-encoding genes in the pathway such as flavanone 3-hydroxylase, flavonoid 3ʹ-hydroxylase and flavonoid 3ʹ,5ʹ-hydroxylase was not induced strongly by UV-C treatment. After 24 h, only some transcripts reached up to 2-fold upregulation.

All the TCs encoding the major secondary metabolite biosynthesis genes and their expression levels in response to UV-C treatment are collected in **Supplementary Table S4**.

### Transcriptional changes in response to CHX treatment

CHX induces *PsSTS* expression (**Fig. 1C**) and in order to choose informative time points for RNA sequencing, we performed a time-series experiment to see how fast CHX was inducing the expression. Increased expression was visible already at 2 h after applying CHX, but strong activation was seen in both replicates after 8 h (**Supplementary Fig. S5**). RNA sequencing was performed on samples treated with CHX for 2, 8 and 24 h in addition to the untreated control. In response to the inhibition of protein synthesis by CHX, 9,851 genes were differentially expressed compared to the untreated control (**Supplementary Table S5**).

**Fig. 3:**
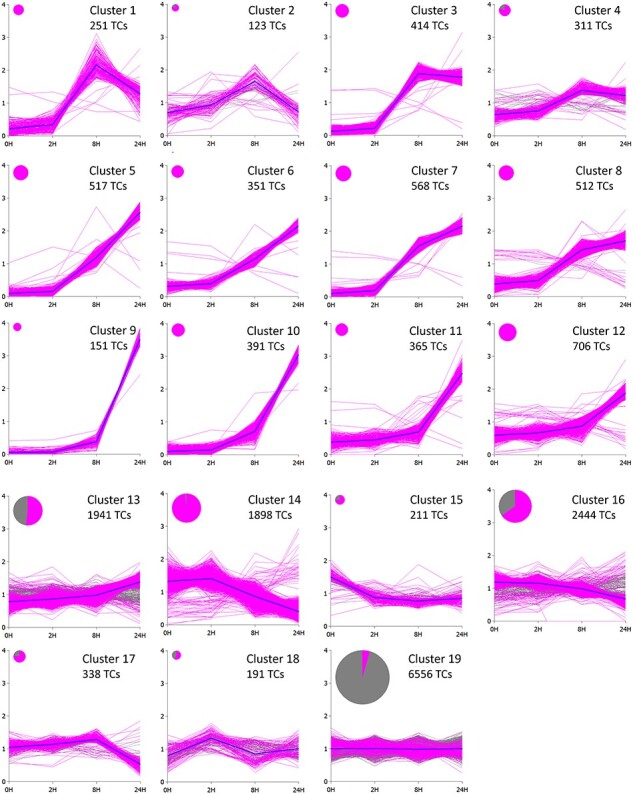
Transcripts from CHX treatment data were grouped by the Bayesian hierarchical clustering algorithm *SplineCluster* (Heard et al. 2006). All TCs that obtained at least eight CPM in at least four libraries are shown. The transcripts that have statistically significant (FDR < 0.05) differences in expression levels compared to the control are shown in color. The area of the disc is proportional to the number of TCs in the cluster. 0H, control; 2H, 2 h; 8H, 8 h and 24H, 24 h.

*SplineCluster* analysis was also performed on the CHX data to discover how genes are responding to the treatment and which genes behaved like PRG (Heard et al. 2006). After mapping against the Pinus EST collection, 18,239 transcripts achieved at least eight CPM in at least four libraries and were kept. These were subjected to hierarchical clustering and were grouped into 19 clusters according to their expression profiles (**Fig. 3**, **Supplementary Table S6**). Responses to CHX can be divided into similar expression patterns as the UV-C data. Clusters 1–4 followed the first pattern where the expression peaked 8 h after the treatment and then declined and, in some cases, returned to the control level. In the second pattern, in Clusters 5–13, transcription was activated after the treatment, but the peak of the expression was not seen within the time points measured. In the third pattern, Clusters 14–17, the expression of transcripts was downregulated. The fourth expression pattern was flat including Clusters 18–19. In total, 36% of transcripts deposited in *SplineCluster* analysis can be considered as PRGs based on the definition and presence in Clusters 1–13.

GO enrichment analysis of CHX-treated seedlings revealed that multiple different processes were enriched. Upregulated clusters had GO terms related to hormonal pathways, such as auxin and jasmonate enriched, which are known to be regulated through labile repressors. In addition, stress responses, metabolism and signaling were all enriched in CHX-treated samples. In downregulated clusters for instance, GO terms associated with amino acid catabolism and general metabolism were downregulated.

### Transcriptional responses of secondary metabolite pathways to CHX treatment

Different secondary metabolism pathways responded differently to CHX treatment. Full-length *PsSTS* and *PsPMT2* genes were found in Cluster 9 following the second pattern and were upregulated but did not reach the peak of expression during the measured time points. Additional transcripts were present also in Cluster 10, following the same pattern. PAL- and 4CL-encoding transcripts were found both in inducible clusters following Pattern 2: 5–7, 10, 11 and 13, and in downregulated Clusters 14 and 16 and non-inducible Cluster 19 of CHX treatment.

The expression of chalcone synthase was downregulated in response to CHX treatment, and the TCs were mostly in Clusters 14 and 16. Plastidic terpenoid biosynthesis–encoding genes involved in the core MEP pathway and downstream genes involved in mono- and diterpene biosynthesis were downregulated. MVA pathway, in contrast, was strongly upregulated by CHX treatment. One specific sesquiterpene synthase–encoding gene strongly upregulated in response to CHX was *E*,*E*-*α*-farnesene synthase, which is present in Cluster 5. Several dirigent proteins involved in lignan biosynthesis were upregulated in CHX data and were present in Cluster 5. Genes specific to the lignin pathway were slightly upregulated in Clusters 12 and 13 or downregulated in Cluster 14. Expression data of secondary metabolite pathway–encoding genes in response to CHX treatment are collected in **Supplementary Table S4**.

### Novel candidates for uncharacterized enzymes of pine stilbene pathway

The stilbene pathway–specific PsPAL and Ps4CL enzymes are not defined in Scots pine. The PsSTS- and PsPMT2-encoding genes (TC154538 and TC166778, respectively) were present in the same clusters after both UV-C and CHX treatments. These clusters, however, did not contain any PAL- or 4CL-encoding genes. Several PAL- and 4CL-encoding TCs were present both in other upregulated and downregulated clusters after both UV-C and CHX treatments. What makes their analysis complicated is that these enzymes are shared between stilbene, lignin, lignan and flavonoid pathways. Different secondary metabolite pathways seemed to respond differently at the transcriptional level to the treatments studied here when specific enzymes of each of the pathways are compared. STS was strongly upregulated, chalcone synthase was downregulated or remaining constant and HCT and CCR from lignin pathway were slightly upregulated. This difference is especially clear in the CHX data. All transcripts encoding PAL and 4CL present in the CHX data were collected and plotted to visualize different expression patterns (**Supplementary Fig. S6**). The best candidates were TC172794 encoding PAL, which was highly upregulated in response to CHX treatment and two 4CL-encoding TCs TC179200 and TC191762 that were induced at similar levels as the characterized PsPMT2 enzyme. Simple blast of known CNL amino acid sequences, from enzymes capable of activating cinnamate in petunia and Aaron’s beard, to pine TC collection did not reveal any similar sequences.

Expression values of the core stilbene biosynthetic pathway enzyme-encoding genes are collected in **Supplementary Table S4**.

### Novel TF candidates for the pine stilbene pathway

This far no regulators of the stilbene pathway in pine have been characterized. To predict what kind of TFs might be involved in regulating the stilbene pathway, we performed an in-silico promoter analysis of the Scots pine *STS* promoter PST-1 (Preisig-Müller et al. 1999) using PlantPan 2.0 (Chow et al. 2016) software to screen putative TF-binding elements. According to Preisig-Müller et al. (1999), the PST-1 promoter has the highest responsiveness of all the *PsSTS* promoters when tested in transgenic tobacco. The promoter contains one or several binding sites for several TF families such as NAC, MYB, WRKY, bZIP, bHLH, AP2/ERF, Homeodomain and GRAS (**Supplementary Table S8**).

**Fig. 4:**
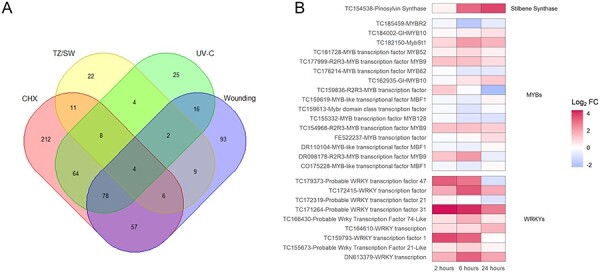
(A) All differentially expressed transcripts associated with GO terms GO:0003700 (sequence-specific DNA-binding TF activity) or GO:0003677 (DNA binding) were extracted from UV-C and CHX RNA-seq data and from previously published TZ/SW (Lim et al. 2016) and wounding data (Lim et al. 2021). A Venn diagram was built from the extracted TF data to see the common regulators in each condition. (B) The expression of MYB and WRKY TFs, the most likely candidates for *PsSTS* transcriptional regulation, from UV-C data was further compared to *PsSTS* expression to reveal co-expressed TFs. The heatmap illustrates the log_2_ fold change of the expression after UV-C treatment compared to the untreated control.

We do not know if in pine stress and developmental regulation of stilbene biosynthesis is mediated by the same TF, as it is in grapevine, via VvMYB14 and VvMYB15 (Höll et al. 2013). To find putative regulators of the Scots pine stilbene pathway, all differentially expressed transcripts associated with GO terms GO:0003700 (sequence-specific DNA-binding TF activity) or GO:0003677 (DNA binding) were extracted from UV-C and CHX RNA-sequencing data and from previously published transition zone/sapwood (TZ/SW) (Lim et al. 2016) and wounding data (Lim et al. 2021), two other conditions where stilbene pathway is activated. TFs from all these different treatments and tissues were compared to find regulators that would be common for both stress and developmental conditions. A Venn diagram was built from the extracted TF data to identify these common regulators (**Fig. 4A**). Only four genes encoding transcriptional regulators were common in all conditions, two WRKYs (TC166430 and TC172319), one ERF (TC157450) and one IAA (TC196936) protein. Two additional TFs, CCAAT-box-binding factor hap3 and NFYB, can be added to the list if we leave CHX treatment out from the comparison. The expression profiles of these genes are shown in **Supplementary Table S9**. *WRKY* TC166430 and *ERF* have inducible expression profiles in all the studied conditions, and the expression of *IAA* is downregulated in the conditions where *PsSTS* expression is the highest, resembling more like a putative repressor. None of the MYB TFs was found to be commonly expressed.

It is possible that the stilbene pathway in Scots pine is regulated via different TFs in different inducible conditions, and by focusing only on commonly expressed genes in all the conditions, we can miss some important regulators of the pathway. Only in the UV-C data, there are 201 differentially expressed TFs from different families. Since R2R3-MYB and WRKY TFs are shown to be the most important TF families to regulate *VvSTS* expression in grapevine, we focused on these families to find additional candidates for future studies. All differentially expressed TFs from MYB (16) and WRKY (9) families were extracted from UV-C data generated in this study, and their expression was compared to *PsSTS* expression (**Fig. 4B**). Three R2R3-MYB (TC177999, TC154966 and DR098178) and four other types of MYB (TC184004, TC182150, TC181728 and TC162935) TFs showed inducible expression patterns in response to UV-C treatment. All of the nine WRKYs showed some level of induction as a response to UV-C treatment, and it is difficult to pinpoint which one(s), if any, could be involved in stilbene pathway regulation. The closest ortholog for grapevine R2R3-MYB14 and 15 and *Picea glauca* MYB12 (ABQ51228) (Höll et al. 2013) in pine is the R2R3-MYB TC182032. This TC was strongly expressed in CHX data and showed an inducible expression pattern in response to UV-C treatment but was filtered out because it did not fulfill the cutoff criterion of eight CPM in at least four libraries. The candidate MYB TF TC188897 that was co-expressed with *STS* in the TZ (Lim et al. 2016) was strongly activated in response to CHX treatment but not in response to wounding or UV-C. The NAC TF (TC164798) that was expressed in the TZ and following expression of pine stilbene pathway enzymes (Lim et al. 2016) was not differentially expressed in any other treatment in addition to TZ. The expression profiles of commonly expressed TFs and MYB candidates that were not present in UV-C data R2R3-MYB TC182032 and MYB TC188897 are shown in **Supplementary Table S9**.

### Induction of STS expression in response to phosphatase inhibitors and plant hormones ethylene and jasmonate

**Fig. 5:**
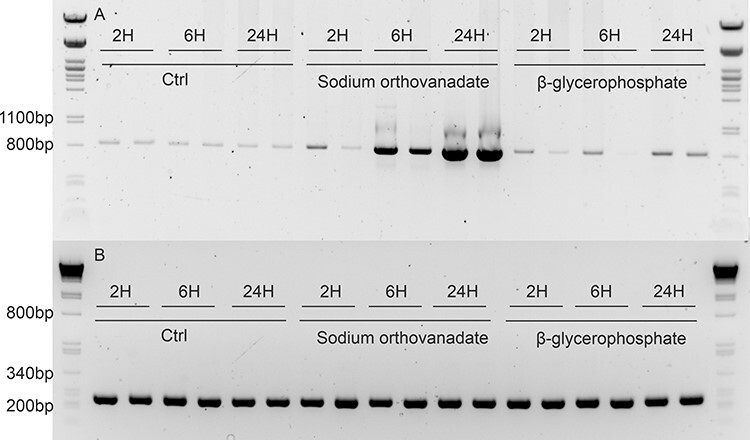
(A) The expression of *STS* in pine needles was analyzed after 2, 6 and 24 h of treatment with 1 mM sodium orthovanadate and 1 mM β-glycerophosphate or from untreated control plants. Strong induction of *STS* gene expression was seen 6 h after the treatment. Slight induction of *STS* was seen 24H of β-glycerophosphate treatment. The expected size of the *PsSTS* fragment is 853 bp. (B) The expression of housekeeping gene *actin* was used as a control.

We wanted to test if the other known inducers of the stilbene pathway in grapevine, the tyrosine phosphatase inhibitor, sodium orthovanadate (Tassoni et al. 2005) and plant hormones jasmonate and ethylene would induce the pathway also in pine (Grimmig et al. 1997, Tassoni et al. 2005, Belhadj et al. 2008). First, the effect of inhibition of protein phosphatases was tested with a broad-spectrum inhibitor cocktail (**Supplementary Fig. S7**). The treatment strongly activated the expression of *PsSTS* with similar kinetics as CHX and UV-C. Next, the inhibiting effect of protein phosphatases was tested using more specific inhibitors targeting tyrosine phosphatases separately with sodium orthovanadate and Ser/Thr phosphatases with sodium pyrophosphate (**Fig. 5**) and β-glycerophosphate (**Supplementary Fig. S8**). Combined effects were tested as well (**Supplementary Fig. S8**). Tyrosine phosphatase inhibitor had a much stronger activating effect on the expression of the STS gene than Ser/Thr phosphatase inhibitors did. Ser/Thr phosphatase inhibitors had only a mild effect on *PsSTS* gene expression.

**Fig. 6:**
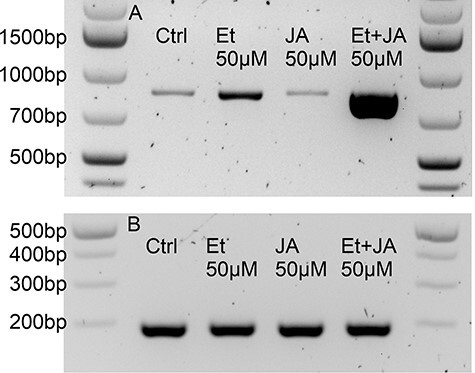
(A) The induction of *PsSTS* in response to treatment with 50 μM ethylene (Et) and 50 μM JA or the combination of both (50 μM Et + 50 μM JA) for 24 h. Ethylene and jasmonate separately caused erratic activation of *PsSTS*, but when applied together, induction was strong and consistent. Each sample has five plants pooled together. In control samples, the hormones were replaced with MQ water or EtOH and collected at the same time as the hormone-treated samples. The expected size of the *PsSTS* fragment is 853 bp. (B) The gene encoding *actin* was used as a control.

We tested the response of the key enzyme of the stilbene pathway, *PsSTS*, to ethylene and JA. Ethylene and JA did not cause a consistent increase in *PsSTS* expression between different experiments and replicates when applied separately, but when applied simultaneously, the induction was strong and repeatable (**Fig. 6**). Interestingly, there are transcripts encoding 1-aminocyclopropane-1-carboxylic acid (ACC) oxidase, an enzyme that converts ethylene precursor ACC into ethylene, present in the same Cluster 8 with *PsSTS* and *PsPMT2*.

### Regulation of the pine STS promoter PST-1 in a non–stilbene-producing plant

**Fig. 7:**
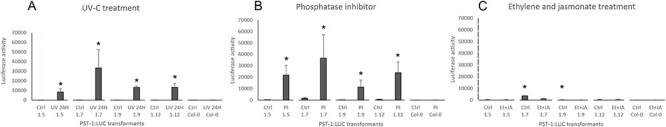
Four individual transformants (1.5, 1.7, 1.9 and 1.12) of Arabidopsis carrying PST-1:LUC promoter–reporter construct were treated with UV-C (A), phosphatase inhibitor cocktail (B) or combination of 50 μM ethylene precursor ACC and 50 μM of jasmonate (C). Plants were sampled before and after the treatment and LUC activity was measured. UV-C and phosphatase inhibitor treatment resulted in increased LUC activity in all the independent transformants. Hormonal treatment had no inducing effect on LUC activity, but in some transformants, slightly higher LUC activity was detected in non-treated control samples. Error bars represent standard errors from three to five replicates. Statistical analyses were performed using a two-sample *t*-test. The treatment group having a statistically significant difference with a non-treated group with *P* < 0.05 is marked with an asterisk.

Scots pine has five genes encoding STS, which were named PST-1 to PST-5 (Preisig-Müller et al. 1999). It was shown that pine stilbene promoters activate reporter gene expression in response to UV-C, wounding and pathogen infection in species that do not normally produce stilbenes such as in tobacco (*Nicotiana tabacum*), where PST-1 showed the highest responsiveness (Preisig-Müller et al. 1999). We transformed Arabidopsis with the PST-1 promoter combined with the firefly luciferase (LUC) reporter gene. We tested if the PST-1 promoter can drive LUC gene expression in response to the same treatments found to induce *PsSTS* gene expression in pine. Clear LUC activity was detected in plants that were exposed to UV-C or treated with phosphatase inhibitors. However, the pine promoter was not able to activate LUC expression in response to combined treatment with plant hormones ethylene and JA (**Fig. 7**).

## Discussion

UV-C treatment was the first discovered artificial stress inducer of the stilbene pathway in pine (Schoeppner and Kindl 1979). A large-scale analysis of transcriptomic responses to UV-C has been utilized in grapevine to study plant responses to the treatment and to discover regulators of the pathway (Vannozzi et al. 2012, Höll et al. 2013, Xi et al. 2014). UV-C responses have not been studied in conifers with RNA-seq before. In this study, we exposed 6-week-old pine seedlings to UV-C treatment and analyzed their needle transcriptomes. Our main aim was to discover co-expressing TFs as candidates for regulating the stilbene pathway in pine and find the pathway-specific cinnamate-activating CoA ligase. In general, UV-C caused massive changes in gene expression in pine with 6,131 differentially expressed TCs in at least one of the analyzed time points. The GO enrichment analysis showed similar responses in pine as seen in grapevine. In brief, stress-response pathways were upregulated and chloroplast and photosynthesis genes were downregulated.

Protein synthesis inhibitors, like CHX, are used for identifying whether the initiation of gene expression requires de novo protein synthesis or is regulated by preexisting TFs (Tullai et al. 2007, Bahrami and Drabløs 2016). The definition of a PRG is that its transcription is initiated without de novo synthesis of transcriptional regulators. Treating plants with translational inhibitor CHX caused even bigger changes in gene expression than UV-C treatment did. In total, 9,851 genes were differentially expressed compared to the untreated control. In total, 36% of transcripts deposited in the *SplineCluster* analysis can be considered as PRGs based on the definition and present in Clusters 1–13 in which the transcription was activated after the treatment. However, we cannot completely rule out the possibility that some of the responses we see for CHX treatment are due to secondary effects of the treatment such as CHX-induced ROS production (Tenhaken and Rübel 1998) or disturbance of the phosphorylation status (Usami et al. 1995) instead of translational inhibition per se. Based on the GO analysis, CHX treatment induced changes to variable processes from stress, metabolism and signaling and affected gene expression heavily.

### Phenylpropanoid pathways respond to UV-C exposure and CHX treatment in different ways

In pine and grapevine, UV-C exposure causes strong transcriptional activation of the stilbene pathway and subsequent accumulation of stilbenes (Vannozzi et al. 2012, Xi et al. 2014, Suzuki et al. 2015). Transcriptional activation of the stilbene pathway in needles was clearly seen in the RNA-seq data, and the product pinosylvin was detected 24 h after the treatment. Several pinosylvin synthase–encoding TCs and an enzyme-encoding gene downstream of the pathway, *PsPMT2*, were upregulated. We saw that the lignin pathway–specific enzymes HCT and CCR were only slightly induced by UV-C, but the flavonoid pathway–encoding genes, such as *CHS*, did not show induction. It is possible that the stilbene pathway is induced at the expense of the flavonoid pathway because they use the same precursors, and this feature seems to be conserved between angiosperms and gymnosperms since, in grapevine, induction of the stilbene pathway suppressed the expression of CHS and biosynthesis of flavonoids (Jeandet et al. 1995, Vannozzi et al. 2012). It has further been shown that in tissues and developmental stages where the flavonoid pathway is active, STS expression is very low (Jeandet et al. 1995, Vannozzi et al. 2012). In *Pinus densiflora*, pinosylvin inhibits CHS enzyme activity (Kodan et al. 2002).

For the pine stilbene biosynthetic pathway, the genes encoding pathway-specific PAL- and cinnamate-activating enzymes are not characterized. In Aaron’s beard and petunia, cinnamate is activated by CNL in the biosynthetic pathway of benzoic acid and its derivatives (Gaid et al. 2012, Klempien et al. 2012). We could not find genes encoding CNL-like enzymes from the Pinus EST collection. Thus, it seems more likely that cinnamate is converted by a specific 4CL variant like in raspberry (Kumar and Ellis 2003). Several PAL- and 4CL-encoding TCs were present in both upregulated and downregulated clusters in both UV-C and CHX treatment transcriptomes. From the phenylpropanoid pathways, only stilbene pathway genes seem to react strongly to CHX treatment, and this might help to find enzymes upstream of STS. From the transcriptomic data, one inducible PAL was discovered. TC172794 encoding PAL was highly upregulated in response to CHX treatment. Two 4CL-encoding TCs TC179200 and TC191762 were induced at similar levels as the gene for the PsPMT2 enzyme. The capability of these enzymes to activate cinnamate needs to be shown in additional experiments.

### Regulators of pine stilbene pathway

Since the stilbene pathway in grapevine and phenylpropanoid and flavonoid pathways in many species are regulated by MYB family TFs, it is the most likely candidate family to regulate the pine stilbene pathway as well (Patzlaff et al. 2003, Bomal et al. 2008, 2013, Falcone Ferreyra et al. 2012, Höll et al. 2013, Wong et al. 2016). In addition, recent data show that WRKY TFs are coregulating (Vannozzi et al. 2018, Wang et al. 2019) or inhibiting (Jiang et al. 2019) *VvSTS* expression, and AP2/ERF are present in the stilbene pathway regulatory network in grapevine (Wong et al. 2016).

The specific TFs regulating the stilbene pathway in pine are not known. In silico promoter analysis of *PsSTS* revealed several putative TF-binding elements in the promoter of pine STS-encoding gene. When we compared the expression of transcriptional regulators in RNA-seq data from developmental heartwood formation and in stress conditions such as UV-C, wounding and CHX, no common MYB TFs for all the conditions were found. Only four genes encoding transcriptional regulators were common in all conditions, two WRKYs, one ERF and one IAA protein. In grapevine, the same MYB TFs, VvMYB14 and VvMYB15, mediate *VvSTS* gene induction and stilbene production both in stress induction when plants were wounded, infected with downy mildew or treated with UV-C and based on developmental cues (Höll et al. 2013). It is possible that in pine different factors are regulating developmental and stress responses or that the TFs are not regulated clearly at the transcriptional level. However, a different set of TF-encoding genes from different families were induced in response to UV-C, and several good candidates were obtained from the experiment. Particularly interesting candidates are the VvMYB 14 and 15 homolog R2R3-MYB (TC182032) and WRKY (TC166430) and ERF (TC157450) TFs expressed in all stilbene-producing conditions. Additional experiments are needed to show which ones, if any, are in pathway.

A further complication for finding the regulators of the pine stilbene pathway is brought by the fact that CHX treatment induces the expression of stilbene pathway genes, which suggests that preexisting transcriptional machinery would already be present in the cells and simply activated in response to the signal. This is a common means to regulate, for instance, hormone-responsive genes, where activating TFs are counteracted by labile repressors. Under CHX treatment, labile repressors cannot be replaced after their degradation and transcription is activated (Moore et al. 2011). In grapevine, VvWRKY8 represses STS gene expression by direct interaction with VvMYB14 and in this way prevents its binding to the promoter (Jiang et al. 2019). We showed that the stilbene pathway genes (*PsSTS* and *PsPMT2*) were transcriptionally activated with slower kinetics than most of the PRGs and resembled this way more delayed PRGs (Tullai et al. 2007). On the other hand, some genes such as JAZ repressors are transcriptionally activated both in response to CHX and treatments such as wounding (Chung et al. 2008), which supports using transcriptional data for finding candidate regulators. So far, there is no experimental evidence of CHX induction of stilbene pathway genes in other stilbene-producing species such as grapevine.

### Many aspects of stilbene pathway regulation are shared between angiosperms and gymnosperms

We know from previous studies that the stilbene pathway is activated in pine, for instance, in response to UV-C, wounding, fungal infection and ozone fumigation, in addition to developmental cues (Schoeppner and Kindl 1979, Lange et al. 1994, Zinser et al. 2000, Harju et al. 2009). The Scots pine STS is encoded by a family of five genes (Preisig-Müller et al. 1999), but we do not yet know if there is a division of labor between the different family members in different conditions as it is in grapevine. With short RNA-seq reads, we could not tell apart the different paralogs. The expression patterns and timing of different members of the large Vitis *STS* gene family vary in response to different stress treatments, which indicates transcriptional subfunctionalization in response to different signaling pathways (Vannozzi et al. 2012).

In general, the mechanisms and components of the signaling pathways under different inductive conditions of pine stilbene biosynthesis are still unknown. It has been suggested that the plant hormone ethylene regulates the biosynthesis of stilbenes also in Scots pine. Treatment of pine sapwood with ethylene was shown to induce the production of pinosylvin and its monomethyl ether (Nilsson et al. 2002). The ethylene biosynthesis gene encoding 1-aminocyclopropane-1-carboxylate oxidase (ACO) was induced in the TZ where the accumulation of stilbenes and conversion of sapwood to heartwood occur. However, the year around expression profile of *ACO* did not support the involvement of ethylene in the stilbene pathway induction during heartwood formation (Lim et al. 2016). In this study, it was shown that treatment of pine seedlings with the ethylene precursor ACC caused only erratic induction of *STS* expression in needles. JA treatment did not induce the *STS* gene expression either, but a combination of these two hormones caused strong and reproducible activation of *PsSTS*. Defense genes such as chitinase (Lorenzo et al. 2003), plant defensin gene *PDF1.2* (Penninckx et al. 1998), pathogenesis-related proteins *PR-1b* and *PR5* (Xu et al. 1994) and proteinase inhibitor (O’Donnell et al. 1996) are examples of defense- and wound-responsive genes where both hormones are needed for activation of the gene expression and in many cases the TF responsible for the regulation is ethylene response factor 1 (ERF1) (Lorenzo et al. 2003). It is possible that JA and ethylene regulate the expression of STS gene in stress-induced conditions but not in the developmental context.

In grapevine, sodium orthovanadate, an inhibitor of tyrosine phosphatase, induced the expression of STS genes (Tassoni et al. 2005). This is another conserved feature in the regulation of stilbene pathways between grapevine and pine. When we disturbed the phosphorylation status of Scots pine cells with protein phosphatase inhibitors, the expression of STS was activated, suggesting that at some level the stilbene pathway is under negative regulation of protein phosphatases. Sodium orthovanadate, a tyrosine phosphatase inhibitor, had a stronger effect on the activation of the transcription than the tested Ser/Thr phosphatase inhibitors. Phosphorylation and dephosphorylation of TFs have been shown to be important mechanisms for initiating the transcription of PRGs. The TFs regulating PRG can be both activated and deactivated by phosphorylation (Moore et al. 2011, Bahrami and Drabløs 2016).

Regulation of the Scots pine STS gene expression is interesting. Even when the *STS* gene has evolved from *CHS* (Tropf et al. 1994), it seems to be under different regulation. STS-encoding genes are responding to CHX treatment, showing features of fast responding primary responses, unlike CHS-encoding genes. When the pine *STS* gene promoter was transformed into Arabidopsis, a species that normally does not synthesize stilbenes, the stress inducibility was retained, but not in all of the studied conditions. UV-C and regulation by phosphorylation were retained but not hormonal regulation. This, and shared features between gymnosperm and angiosperm regulation, indicates that STS regulation occurs not only via ancient stress-response pathway(s) but also via species-specific regulators.

## Conclusion

Stilbenes are important metabolites for the biotic and abiotic stress tolerance and durability of pine heartwood. To efficiently breed more resistant trees using DNA markers, we need to know the genes involved in the biosynthesis and regulation of the stilbene pathway in pine. The differences in these genes can be the source for the observed variation in the stilbene production capability between the individuals. In this study, new candidates for specific regulatory TFs, as well as genes encoding putative PAL- and 4CL-like-encoding genes specific to the stilbene pathway, were identified. Furthermore, we showed that the pine stilbene pathway is regulated by protein (tyrosine) phosphatases and synergistic action of plant hormones ethylene and jasmonate, both influencing stilbene pathway expression in grapevine as well. Both kinases and phosphatases that are needed for the pathway regulation are still unidentified and need to be studied in the future. Since pine stilbene promoter retains its inducibility in Arabidopsis, a species that do not naturally produce stilbenes, some conserved regulators and regulation mechanisms involving phosphorylation/dephosphorylation events seem to be conserved between gymnosperms and angiosperms. Further studies are needed to identify these common stress regulators.

## Materials and Methods

### Chemicals

All chemicals were purchased from (Sigma-Aldrich, Burlington, Massachusetts, United States) unless stated otherwise.

### PST-1 promoter–LUC reporter vector construction and Arabidopsis transformation

DNA was isolated from pine needles using the CTAB method as described by Michiels et al. (2003). The PST-1 promoter was amplified from genomic DNA with primers attB4 PST-1F and attB1r PST-1R (**Supplementary Table S10**). The PCR product was inserted into pDONRzeoP4P1r vector using Gateway BP clonase enzyme (ThermoFisher Scientific, Waltham, Massachusetts, United States) according to the manufacturer’s instructions. Firefly LUC gene with an intron (Mankin et al. 1997) was first amplified from plasmid pLKB10 with primers attB1 FLUC-F and attB5 FLUC-R, and the PCR product was further amplified with primers Adapter attB1 and Adapter attB2 to generate the full attB1 and attB2 attachment sites. This PCR product was then inserted in pDONR221 vector using the Gateway BP reaction. Multisite Gateway cloning was performed using LR clonase II Plus enzyme to fuse the PST-1 promoter fragment (attL4-attR1sites), the LUC fragment (attL1-attL2 sites) and a nopaline synthase terminator with attR2-attL3 sites to the pCAMkan-R4R3 destination vector. The final reporter construct PST-1:LUC:NOS was then transferred to Arabidopsis Columbia-0 ecotype using agrobacterium GV3101(pMP90) and the floral dip method (Clough and Bent 1998). Three rounds of selection were performed in MS plates having 50 μg/ml of kanamycin. First, primary transformants were selected. T1 lines with one insertion were selected by looking for 1:3 segregation. T2 lines lacking wild-type segregants were propagated to establish homozygous T3 lines. Four independent transformants were used in all experiments.

### Plant growth conditions

For UV-C and chemical treatments, pine seedlings were grown for 6 weeks and Arabidopsis for 3 weeks at 23°C in peat:vermiculite (1:1) under 16 h light and 8 h dark in controlled growth chambers. For the RNA-sequencing experiments, the Scots pine seeds originated from Natural Resources Institute progeny trial from Leppävirta (62°25′ N and 27°45′E), and for the other experiments, the seeds were a gift from Siemen Forelia (Rovaniemi, Finland) and were collected from Hartola (61°36′N and 26°16′E).

### UV-C treatment

For the UV-C experiment, pine and Arabidopsis seedlings were treated for 15 min, at a distance of 20 cm, under an uncovered mercury lamp (sterilAir UVC G9, SterilAir AG, Weinfelden, Switzerland). Spectral photon irradiance (μmol m^−2^ s^−1^) transmitted by the lamp was measured with an array spectroradiometer, which had been calibrated for measurements of UV and visible radiation (Maya2000 Pro Ocean Insight, Dunedin, FL, USA; D7-H-SMA cosine diffuser, Bentham Instruments Ltd, Reading, UK). The main peak from the spectrum was at 254 nm (**Supplementary Fig. S11**), and the irradiance at the UV-C region was 1,396 μmol m^−2^ s^−1^.

The preliminary screen for pine UV responses was performed with single seedlings. Transcriptomes were sequenced from control samples and after 2, 6 and 24 h after the onset of the UV-C treatment. Each sequenced sample had needles from 10 seedlings pooled together, and each time point had four replicate samples (seedlings from open-pollinated mother tree lines T089, T170, T434 and T474). Samples were frozen in liquid nitrogen and stored at −80°C.

For Arabidopsis, five plants from four independent transformants were treated with UV-C for 15 min. Samples for LUC measurements were taken before and 24 h after the treatment. The effect of cutting of the leaf was tested by sampling the same non-treated plants 24 h after the first sampling (**Supplementary Fig. S10A**).

### CHX treatment

For all CHX treatments, seedlings were removed from the soil, and the roots were rinsed with water. Roots were rolled around a pipette tip and seedlings were placed in 1.5-ml microcentrifuge tubes. For the preliminary screen (**Fig. 1**), single plants were treated for 16 h with 10 µM CHX in 1 ml of Milli-Q (MQ) water in microcentrifuge tubes, and the UV-C exposure was performed the following day.

To test how efficiently protein translation was inhibited, ^35^S-methionine was fed to the plants through roots. Forty microliters of 10 mCi/ml EasyTag™ Methionine, L-[35S] (PerkinElmer, Waltham, Massachusetts, United States), were combined with 20 µl of MQ water. Seedlings were placed in microcentrifuge tubes, and 10 µl of mixture was given to dry roots. Every hour, 100 μl of MQ water was added to prevent seedlings from drying out. Treatment was performed for 4 h to two control plants and to four plants pre-treated with 10 µM or 100 µM of CHX for 16 h. Samples were frozen in liquid nitrogen and stored at −80°C. Proteins were extracted from the needles as described by Fliegmann et al. (1992) and were precipitated with one-fourth volume of 100% (w/v) trichloroacetic acid from part of the sample, and the pellet was washed twice with cold acetone. Radioactivity was measured from the extract and the trichloroacetic acid precipitated protein pellet for 5 min with a scintillation counter (Wallac 1414 WinSpectral v.1.40; Wallac Oy, Turku, Finland) using 1 ml of Ultima Gold™ (PerkinElmer) liquid scintillation cocktail. Labeling of newly formed polypeptides showed a reduction of 94% of protein synthesis in samples pre-treated with CHX compared to water control.

For CHX RNA-seq analysis, ten 6-week-old seedlings were combined for each time point and placed in 10 ml of MQ water supplemented with commercial plant fertilizer (Substral, Scotts, Marysville, Ohio, United States) and allowed to recover overnight. The next day, water was replaced with 10 ml of 10 µM CHX dissolved in MQ water or 10 ml of MQ water for the controls. Needles were collected 2, 4, 6, 8, 10 and 24 h after the initiation of the treatment. Control samples were collected at time points 2 and 24 h. The following samples were sequenced from two biological replicates (Lines T392 and T432) using SOLiD platform: control (24 h in water), 2 h after the onset of the CHX treatment for early responses, 8 h when STS expression was clearly up in both replicates and 24-h samples when the expression of STS was at its highest. Samples were frozen in liquid nitrogen and stored at −80°C.

### Other chemical treatments

For hormone, phosphatase inhibitor and anisomycin treatments, pine and Arabidopsis seedlings were removed from the soil and placed in 1 ml of water and allowed to recover overnight. The next day, plants were treated with either 50 µM of ACC, 50 µM of JA or both combined, and samples were collected 6 and 24 h (**Supplementary Fig. S9**) or after 24 h (**Fig. 6**) after the initiation of the treatments. The ACC stock was dissolved in water and JA in ethanol, and controls were treated with a similar volume of ethanol without hormones. Five plants were pooled together for each sample for the pine experiment. Samples were frozen in liquid nitrogen and stored at −80°C. For Arabidopsis, five plants were treated from each line. Plants were sampled before and 24 h after the treatment, and LUC activity was measured directly from fresh samples.

The broad-spectrum phosphatase inhibitor (PhosSTOP, Phosphatase Inhibitor Cocktail Tablet, Roche, Basel, Switzerland) was dissolved in MQ water, and 1x solution was used for the treatments. Treatment was performed for a single pine seedling per time point and five Arabidopsis plants per each line. The tyrosine phosphatase inhibitor sodium orthovanadate was prepared in alkaline water according to the manufacturer’s information. Ser/Thr inhibitors β-glycerophosphate and sodium pyrophosphate stocks were prepared in MQ water. Treatment was performed with 1 mM of inhibitor solutions or alkaline MQ water and pH 10 for controls. Samples were frozen in liquid nitrogen and stored at −80°C for pine samples, and Arabidopsis plants were sampled before and 24 h after the treatment and LUC activity was measured directly from fresh samples.

For anisomycin treatment, inhibitor stock was dissolved in MQ. After the recovery period, MQ was replaced with 10, 25, 50 or 100 µM anisomycin and MQ water for the control plants. Samples were frozen in liquid nitrogen and stored at −80°C.

### Extraction of total RNA and semi-quantitative RT-PCR

Total RNA was extracted from needles as described by Chang et al. (1993) and dissolved in 50 µl of MQ water. RNA was purified and genomic DNA was digested with RNeasy Plant Mini Kit (Qiagen, Venlo, The Netherlands) according to the manufacturer’s protocols. The RNA quality was assessed with agarose gel electrophoresis. First-strand cDNA was synthesized with SuperScript III reverse transcriptase (Invitrogen, Carlsbad, California, United States) according to the manufacturer’s instructions using 500 ng of total RNA as a template. The STS transcript was amplified with primers STS_F and STS_R in all RT-PCR experiments. The size of the product is 853 bp. Control reactions amplifying the housekeeping gene actin were performed with primers Actin_F and Actin_R. PCR reactions were performed using Taq DNA polymerase (Roche Life Science, Basel, Switzerland or Thermo Scientific, Waltham, Massachusetts, United States) according to the manufacturer’s recommendations and using 1 µl of cDNA as a template with the following program: 94°C for 2 min, 24 cycles of 94°C for 30 s, 57°C for 1 min and 72°C for 1 min followed by 72°C for 7 min.

### LUC assay

LUC activity was measured in Arabidopsis by sampling one to two leaves before and after the treatment. Proteins were extracted on ice with 100 μl of modified lux buffer (Nilsson et al. 1992) and microcentrifuge tube pestles. Cell debris was removed by centrifugation for 10 min at +4°C. LUC activity was measured by combining 20 μl of sample with 50 µl of enzyme substrate (Luciferase 1000 Assay System, #E4550, Promega, Madison, Wisconsin, United States), vortexing sample briefly and counting photons for 5 s in the luminometer (Luminoskan TL plus, generation II, Thermo Labsystems, Philadelphia, Pennsylvania, United States) at room temperature. The total protein concentration was measured from samples with Bradford assay at 595 nm using BSA as a standard. Measured LUC activity was normalized using the total protein concentration.

### Thin-layer chromatography

Needles from 6-week-old seedlings were treated for 15 min of UV-C, at a distance of 20 cm under an uncovered mercury lamp (sterilAir UVC G9) and ground to powder under liquid nitrogen. Metabolites were extracted with 5 ml of methanol per gram wet weight, sonicated for 15 min in a water bath sonicator (Finnsonic W181) and left at +4°C overnight. Cell debris was removed with centrifugation and 2 + 2 µl of extracts and a commercial pinosylvin standard (ArboNova, Turku, Finland) were applied on thin-layer chromatography (TLC) plates Silica gel 60 F254 (Merck, Rahway, New Jersey, United States), which were developed with chloroform:ethyl acetate:formic acid (5:4:1) as the mobile phase. Plates were photographed under UV light using 304 nm filter.

### RNA-sequencing analysis

RNA-seq libraries were prepared, sequencing was performed and data analysis was performed as described by Lim et al. (2016, 2021). In short, all transcriptome libraries were mapped against Pinus EST collection version 9.0 (The Dana-Farber Cancer Institute gene index databases, 2014) using SHRiMP alignment tool (David et al. 2011). Pinus EST collection is composed of TC sequences that are assemblies from highly similar sequences from different pine species. For instance, TC154538 for STS is 98% identical to the GenBank Scots pine STS mRNA sequence S50350 (Lim et al. 2016). Differential expression analysis was performed using edgeR (Robinson et al. 2010). When performing the differential expression analysis of UV-C and CHX data, sample similarity was verified by plotting the samples on a two-dimensional scatterplot so that distances on the plot approximate the typical log2 fold changes between the samples. This was performed using edgeR’s MDS. The plots are shown in **Supplementary Fig. S4**. Bayesian model–based hierarchical clustering was performed using *SplineCluster* (Heard et al. 2006). The RNA-seq reads have been submitted to National Center for Biotechnology Information (NCBI) under Bioproject PRJNA635551.

### In silico promoter analysis, GO enrichment and intersect analysis

The Scots pine STS promoter sequence (PST-1) (Preisig-Müller et al. 1999) was submitted to the plant promoter analysis database PlantPan 2.0 (Chow et al. 2016) to screen for putative TF-binding elements. All differentially expressed genes from *SplineCluster* analysis in CHX and UV-C treatments were grouped into four patterns according to their expression profiles through time after treatment. GO enrichment analysis was performed for each pattern using the whole annotation of detected ESTs in each treatment as a reference set, and the GO categories molecular function, biological process and cellular component were analyzed. GO enrichment analysis was performed using Biological Networks Gene Ontology (BiNGO) tool with a hypergeometric test to assess the statistical significance of GO terms Maere et al. 2005). Benjamin and Hochberg's FDR correction was selected for multiple comparisons with a *P*-value threshold of 0.05. All differentially expressed transcripts associated with GO terms GO:0003700 (sequence-specific DNA-binding TF activity) or GO:0003677 (DNA binding) were extracted from UV-C and CHX RNA-seq data and from previously published TZ/SW (Lim et al. 2016) and wounding (Lim et al. 2021) RNA-seq data. Intersection analysis between differentially expressed genes in CHX, UV-C, TZ/SW and wounding treatments was performed using the venn R package.

## Supplementary Material

pcad089_Supp

## Data Availability

RNA-seq data have been submitted to the National Center for Biotechnology Information Sequence Read Archive (http://www.ncbi.nlm.nih.gov/sra) with the BioProject ID PRJNA635551.
