# Supplementary material for: Transcriptomic Analysis Reveals Novel Regulators of the Scots Pine Stilbene Pathway
Source: Plant Cell Physiol. 2023 Aug 9;64(10):1204–19. doi: 10.1093/pcp/pcad089 (PMC10579783; doi:10.1093/pcp/pcad089)
Supplement: pcad089_Supp [file pcad089_supp.zip › suppl_data/pcp-2023-e-00166-File009.pdf]

Transcriptome analysis reveals novel regulators of the Scots pine stilbene pathway

Tanja Paasela, Kean-Jin Lim, Mirko Pavicic, Anni Harju, Martti Venäläinen, Lars Paulin, Petri Auvinen, Katri Kärkkäinen and Teemu H. Teeri

Supplemental figures

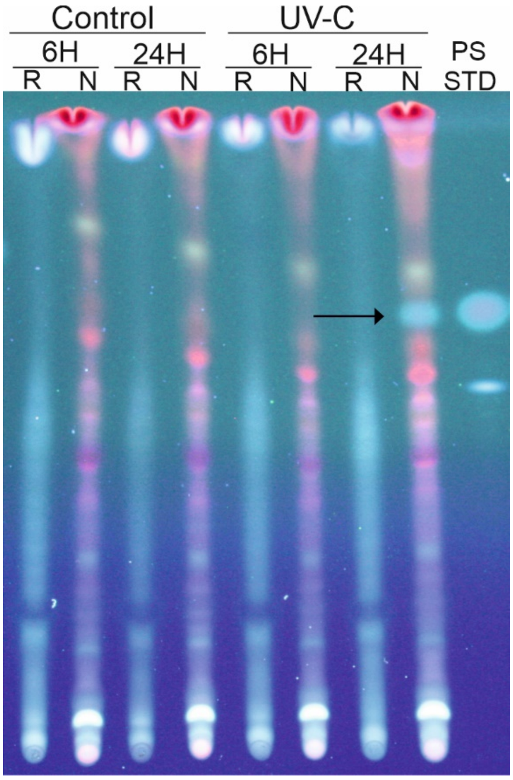

Figure S1. Six-week-old pine seedlings were treated with UV-C and metabolites were extracted from needles (N) and roots (R) with methanol and separated on TLC plate. Pinosylvin (arrow) was formed at 24H after the induction in needles but not in roots. Commercial pinosylvin (PS Std) was used as a standard. The fluorescence of pinosylvin was recorded under 304 nm UV illumination.

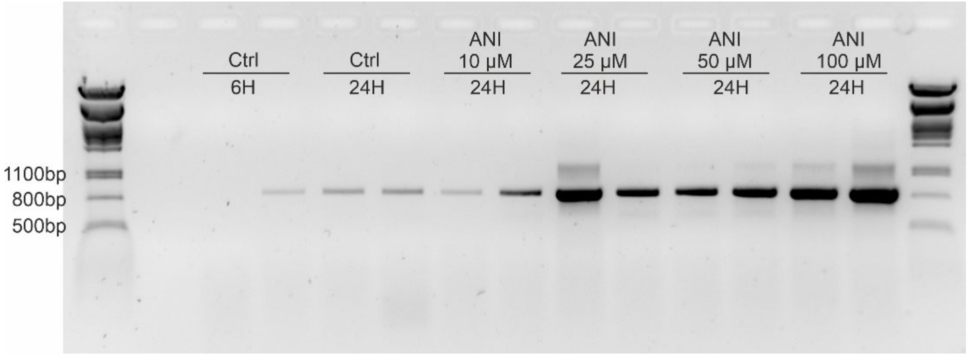

Figure S2. Six-week-old seedlings (two for each treatment) were treated with different concentrations of protein synthesis inhibitor anisomycin (ANI). *PsSTS* gene expression was activated 24 hours after initiation of the treatment. Expected size of the *PsSTS* fragment is 853 bp

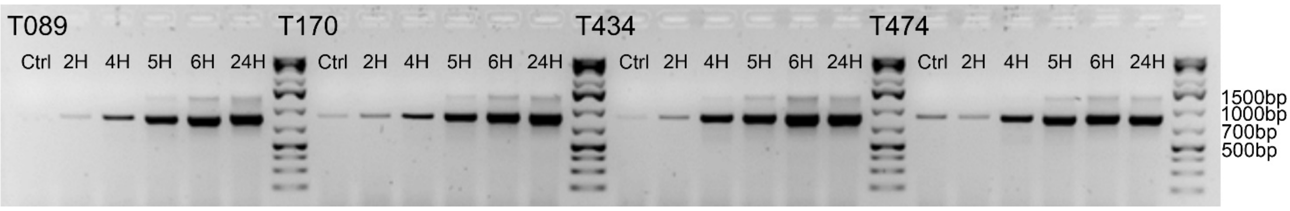

Figure S3. Six-week-old seedlings were treated with UV-C. Expression of *PsSTS* was followed for 24 hours. In each sample ten needles from seedlings were pooled together. Transcriptomes after two, six and 24 hours of treatment and from the untreated control were sequenced from all four replicates (T089, T170, T434 and T474) using the SOLiD platform.

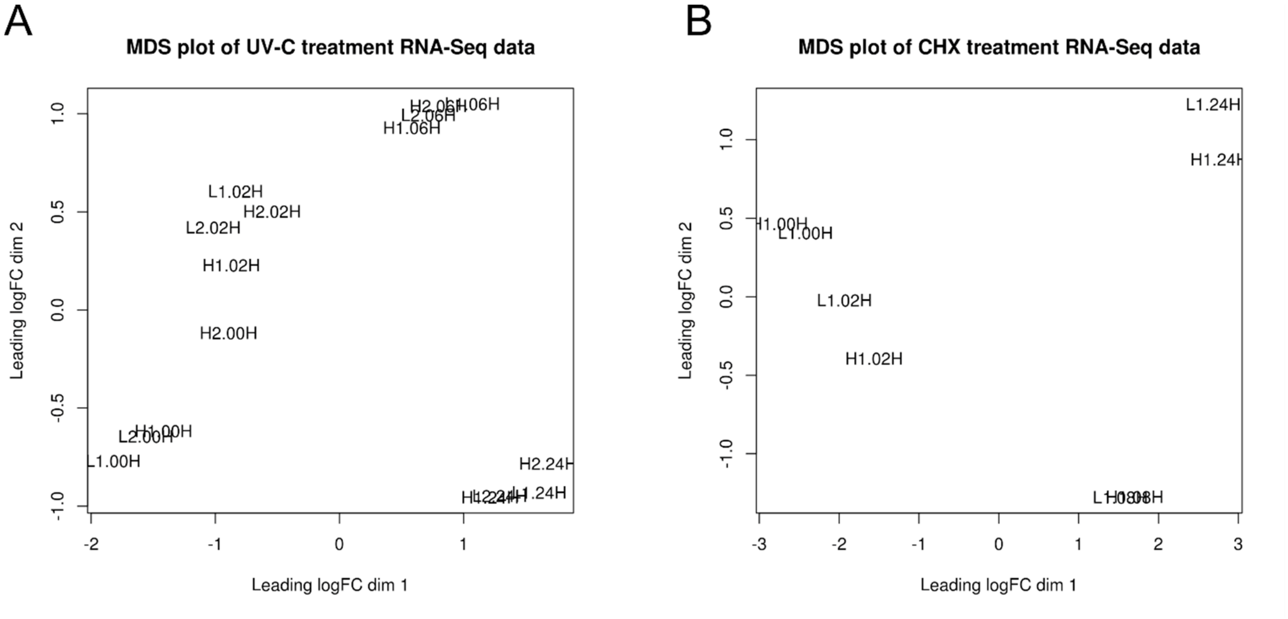

Figure S4. Similarity of replicate samples were tested using MDS plotes. The samples were plotted on a two-dimensional scatterplot so that distances on the plot approximate the typical log2 fold changes between the samples. Different samples grouped together based on the time after treatment. .00H are non-treated samples, .02H samples are collected two hours, .06H six hours, .08H eight hours and .24H 24 hours after the treatment. A) shows analysis from UV-C treated samples and B) shows analysis from CHX treated samples.

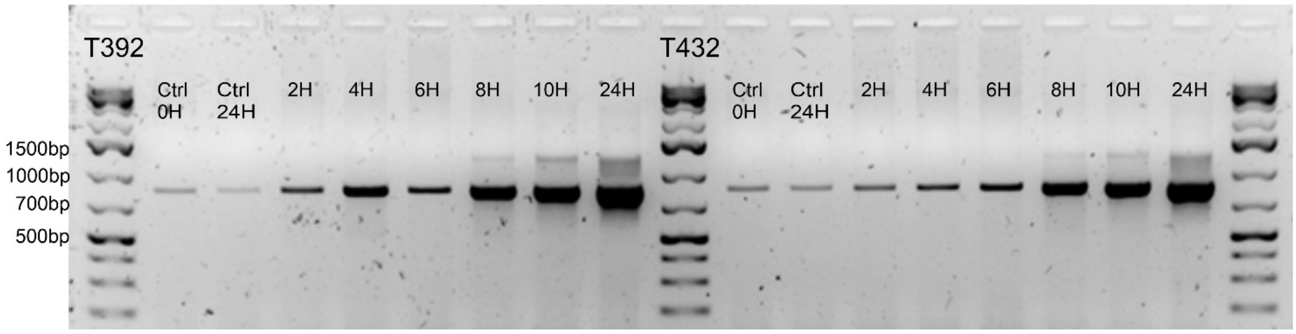

Figure S5. Six-week-old seedlings were treated with 10 µM of CHX. Expression of *PsSTS* was followed for 24 hours. In each sample needles from ten seedlings were pooled together. Transcriptomes after two, eight and 24 hours of treatment and from untreated control were sequenced from two replicates (T392 and T432) using the SOLiD platform.

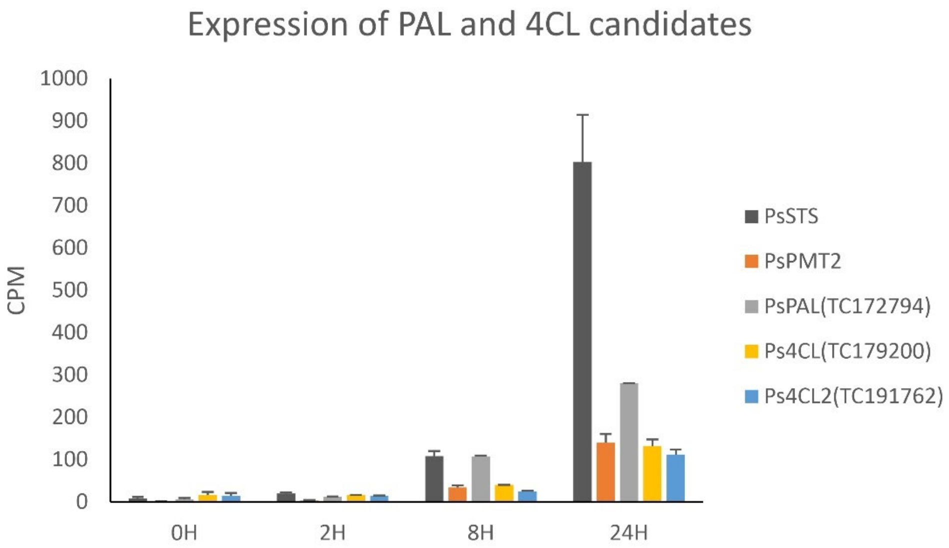

Figure S6. The expression of known stilbene pathway encoding genes PsSTS and PsPMT2 show inducible expression pattern in response to CHX treatment. From the CHX transcriptomic data one inducible PAL and two 4CL (TC179200 and TC191762) were discovered. Their expression is induced at similar levels after CHX treatment as characterized PsPMT2 enzyme.

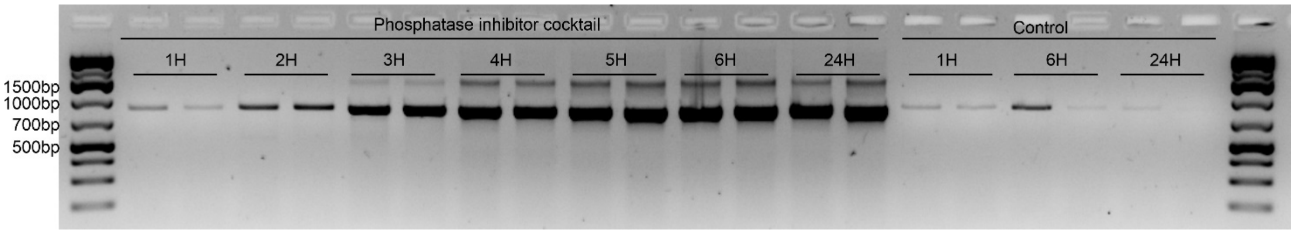

Figure S7. *PsSTS* expression is induced in response to treatment with general protein phosphatase inhibitor. Expression was followed for 24 hours.

Fig S8.

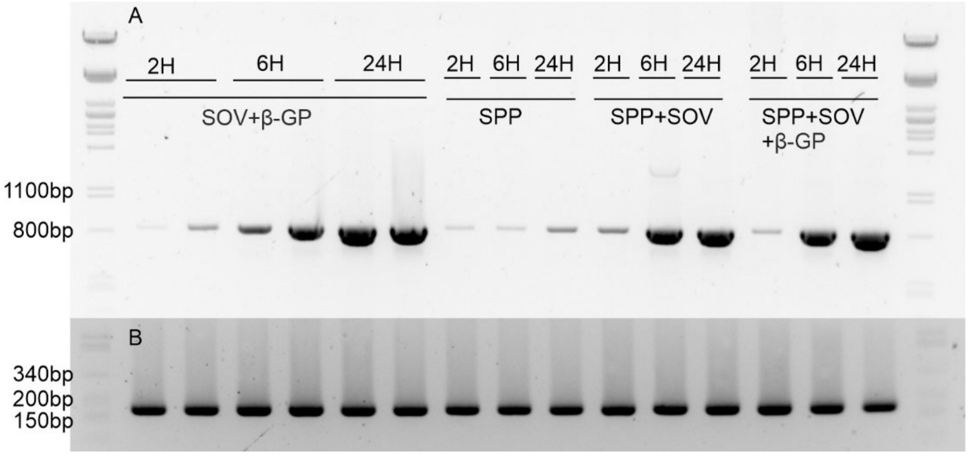

Figure S8. A) The expression of *PsSTS* in pine needles was analyzed after two, six and 24 hours of treatment with different combinations of 1mM sodium orthovanadate (SOV), 1mM β-glycerophosphate (β-GP) or 1mM sodiumpyrophosphate (SPP). Untreated plants were used as a control and shown in figure 2. B) The expression of housekeeping gene actin was used as a control for cDNA quality.

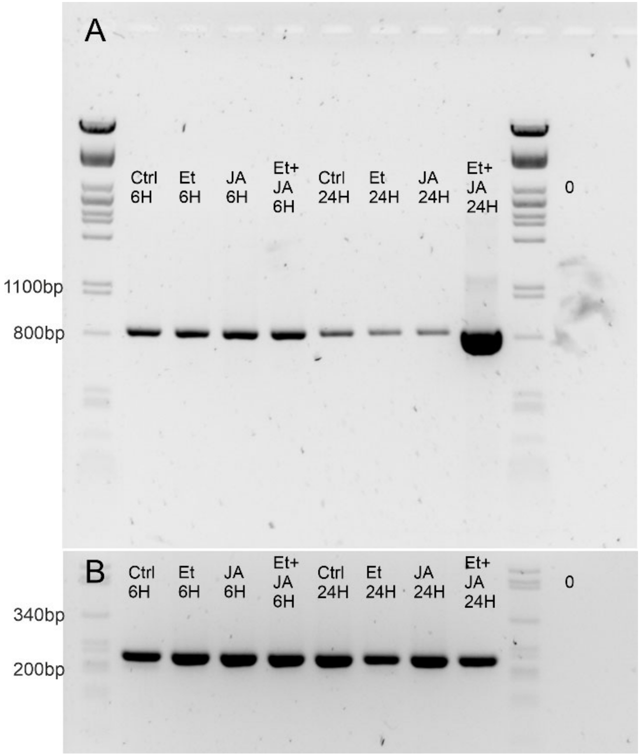

Fig S9. The expression of *PsSTS* in pine needles was analyzed after treatment with 50 µM ethylene (Et) and 50 µM jasmonic acid (JA) or with the combination of both (50 µM Et+50 µM JA) for 6 or 24 hours. Each sample has three plants pooled together. In the control samples the hormones were replaced with MQ water or ethanol. B) The expression of housekeeping gene *actin* was used as a control.

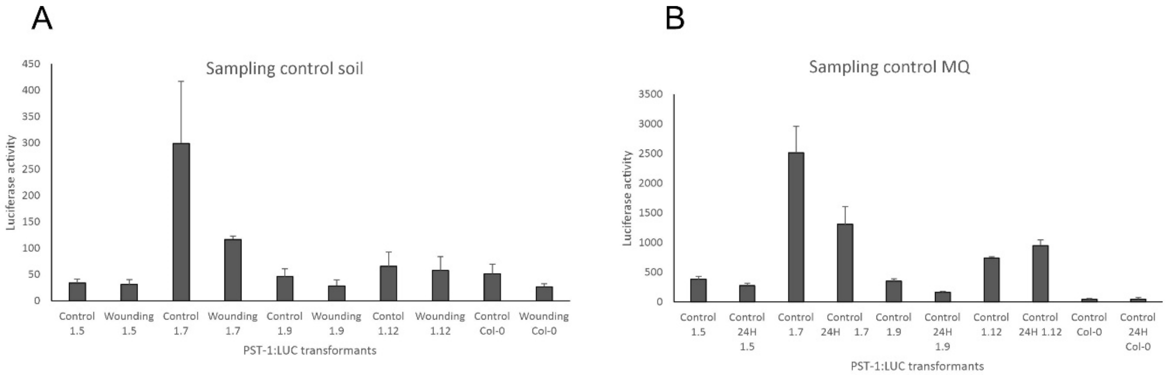

Figure S10. To see if luciferase gene is activated in Arabidopsis PST-1:LUC lines in response to sampling of leaves, plants were re-sampled 24 hours (24H) after the first sampling time without any additional treatment. Plants were either grown on soil (A) as in UV-C experiment or removed from soil and placed in MQ water (B) as in phosphatase inhibitor and hormone treatments. Cutting of leaves did not activate luciferase gene expression in either of the controls.

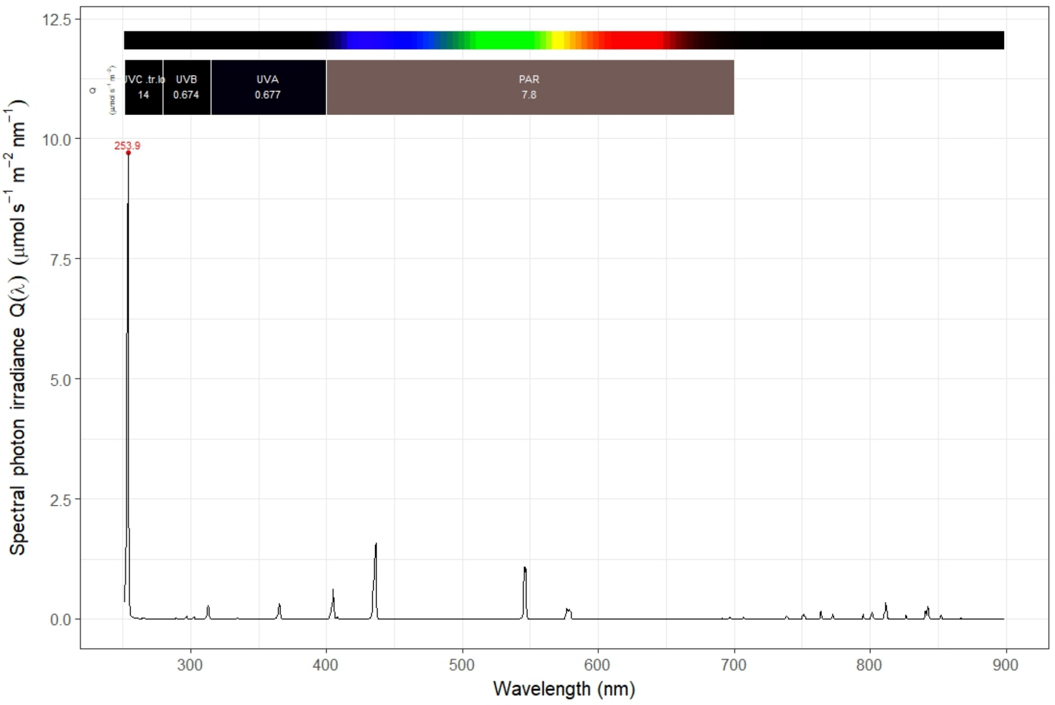

Figure S11. UV-C treatment of seedlings was done with uncovered mercury lamp. The intensity spectrum was measured at the distance of 20 cm from the lamp with an array spectroradiometer calibrated for UV and visible solar radiation. The spectrum had the main peak at 254 nm UV-C region.
